# Long-Term Aspirin Administration Has No Effect on Erectile Function: Evidence from Adult Rats and Ageing Rat Model

**DOI:** 10.1038/s41598-019-44386-x

**Published:** 2019-05-28

**Authors:** Tao Li, Changjing Wu, Fudong Fu, Wenfeng Xiong, Feng Qin, Jiuhong Yuan

**Affiliations:** 1 The Andrology Laboratory, West China Hospital, Sichuan University, Chengdu, Sichuan China; 2 Department of Urology, West China Hospital, Sichuan University, Chengdu, Sichuan China

**Keywords:** Sexual dysfunction, Urogenital diseases

## Abstract

As the broad spectrum pharmacological action, aspirin has been one of the most widely used medicines since its initial synthesis; however, the association between aspirin and erectile function is still controversial. We aim to explore whether long-term aspirin administration deteriorates or preserves erectile function from adult rats and ageing rat model. Twenty adult rats (10 weeks of age) and twenty ageing rats (80 weeks of age) were randomly divided into four groups as follows: Adult-Control (normal saline [NS]), Adult-Aspirin (aspirin, 10 mg/kg/d), Ageing-Control (NS), and Ageing-Aspirin (aspirin, 10 mg/kg/d) groups (n = 10 per group). For all rats, erectile function was assessed by maximum intracavernous pressure (ICP), total area under ICP curve (AUC), ICP/mean arterial pressure (MAP) ratio, and MAP. The total treatment duration was one month. Protein expression levels of cyclooxygenase-1 (COX-1), COX-2, endothelial nitric oxide synthase (eNOS), and nNOS of the corpus cavernosum were detected by Western blot. ELISA kits were used to determine 6-keto PGF_1a_, PGE_2_, TXB_2_, cyclic adenosine monophosphate (cAMP), and cyclic guanosine monophosphate (cGMP) levels. Total nitric oxide (NO) concentration was measured using a fluorometric assay kit. As a result, Ageing-Control rats revealed significantly decreased ICP, AUC, and ICP/MAP ratios compared to Adult-Control rats, and these effects were accompanied by reduced eNOS protein expression and lower total NO and cGMP levels; however, no difference was found in nNOS protein expression. For adult rat groups, aspirin significantly inhibited the production of 6-keto PGF_1a_, PGE_2_, and TXB_2_; however, it neither changed the ICP, AUC, or ICP/ MAP ratios nor altered the protein expression of eNOS, nNOS, COX-1, and COX-2. Meanwhile, aspirin did not influence the concentrations of total NO, cAMP, or cGMP. The same tendency was also found in the ageing rat model, which confirmed that aspirin did not alter erectile function. Our data suggested that long-term aspirin administration did not strengthen or weaken erectile function in adult rats or ageing rat model. Thus, it had no impact on erectile function.

## Introduction

Erectile dysfunction (ED) is defined as the inability to attain or maintain sufficient penile erection for satisfactory sexual performance^1,2^. It is a common condition that affects 31–52% of men aged >50 years^3^ and impairs people’s quality of life^4^. As medicine-induced ED leads to noncompliance with medical prescriptions, it is important to examine the association between medications and ED^4^.

Since its initial synthesis 100 years ago, aspirin has been one of the most widely used medicines^3^ for its broad spectrum indications. Aspirin is now available without prescription for arthritis, joint pain, muscle aches and chronic musculoskeletal pain; it is well known as an important prophylaxis for CVD and atherosclerotic disease^3,5^; other ‘magical’ effects are being explored, including a protective role against stroke, thrombosis, and cancer progression^5–7^. Thus, aspirin usage is common in the older population. Meanwhile, it is also popularly used for any type of febrile condition, acute or chronic pain, and dysmenorrhea^3,5^. As a consequence, it has been the single most important self-prescribed medicine for any aged patient, while the annual drug usage is tremendous. In addition, the usage of aspirin is also accompanied by many adverse effects, like gastrointestinal injury, cerebral bleeding, and anaphylaxis^8–10^; however, its potential impact on erectile function is still controversial^8^.

First, ED shares similar risk factors to cardiovascular diseases (CVD), such as ageing^11,12^, hypertension, diabetes, and hyperlipidaemia^13^. ED has been associated with endothelial dysfunction^2,3,5,7^ and is considered an early predictor of CVD^13,14^. With an excellent protective role against CVD, aspirin should be beneficial for ED. Meanwhile, low-grade inflammation processes or highly circulating pro-inflammatory markers are also related to the ED process^3,15^; it is rational to use aspirin as a preventive treatment against ED, considering its anti-inflammatory abilities^3^.

In contrast, aspirin inhibits the cyclooxygenase (COX) pathway to decrease vasodilative agents of prostaglandin I_2_ (PGI_2_) and prostaglandin E_2_ (PGE_2_)^6^. Considering their vasodilation effects, intracavernous or intraurethral injection (PGE_1_)^1,8,16^ and COX-2-linker-PGIS gene therapies^17^ have been used for penile rehabilitation. Therefore, aspirin should deteriorate normal erectile function, as it reduces these vasodilative prostaglandin agents^4,5^.

In addition, some recent studies revealed that aspirin had no impact on erectile function^2,3,18^. These studies claimed that, similar to arthritis, joint pain, muscle aches, chronic musculoskeletal pain, or atherosclerotic disease, most medical indications of aspirin were also risk factors for ED^3,4^. Therefore, it was necessary to clarify whether these associations were attributed to aspirin or the disease condition itself  ^3^. However, these claims were not supported by any basic evidence which might help to elucidate this relationship^19^.

Considering the broad spectrum indications and tremendous annual drug prescriptions for aspirin, we investigated the relationship between aspirin and erectile function. As more recent clinical studies report that aspirin does not affect erectile function, the positive or negative associations reported may potentially be the result of confounding by indication bias; thus, we also hypothesized that aspirin administration has no impact on erectile function. Moreover, we tested this hypothesis on both adult rats and ageing rat model, considering that aspirin is commonly taken by both adult and aged populations.

## Materials and Methods

### Animals

The experimental male Sprague-Dawley rats were purchased from Dashuo Experimental Animal Co. Ltd., Chengdu, Sichuan Province, China. We confirmed that all experiments were performed in accordance with relevant guidelines and regulations; all rats were housed and cared for under strict guidelines, and this study was approved by the Animal Ethics Committee of West China Hospital, Sichuan University (NO. 20160461A).

Specifically, twenty healthy adult rats (10 weeks of age) and twenty ageing rats (80 weeks of age) were randomly divided into four groups (n = 10 per group) as follows: Adult-Control (normal saline [NS]), Adult-Aspirin (aspirin, 10 mg/kg/d), Ageing-Control (NS), and Ageing-Aspirin (aspirin, 10 mg/kg/d). The total treatment duration was one month.

### Erectile function measurement

With a one-week washout period after the treatment duration, erectile function was assessed in all rats (n = 10 per group) by recording the maximum intracavernous pressure (ICP) and ICP/mean arterial pressure (MAP) ratio according to a previous method^20,21^. Briefly, anesthesia induction was first given at a concentration of 5% (volume/volume) isoflurane (RWD Life Science, Guangdong Province, China) mixed with air^20,22^. The isoflurane concentration was then downgraded and maintained at 2%, while the left carotid was carefully exposed and cannulated with heparinized (200 IU/ml) detaining venipuncture (26 G; Closed IV Catheter System, Becton Dickinson Medical Devices Co. Ltd., Franklin Lakes, NJ, USA) connected to a pressure transducer to measure arterial pressure. The left cavernous nerve was carefully exposed and isolated after a low midline abdominal incision was made. After the penis was denuded of skin, a heparinized (200 IU/ml) 24-gauge needle (SGJS Medical Equipment Group Co. Ltd., Luohe, Henan Province, China) connected with a BL420 bio-function experiment system (Chengdu TME Technology Co. Ltd. Chengdu, Sichuan Province, China) was inserted into the penile crus to record the ICP. The concentration of isoflurane was then decreased to 1%, and the cavernous nerve was electrically stimulated as follows: using 2.5 V, 5 V, 7.5 V at a frequency of 20 Hz, pulse width of 5 millisecond and duration of 60 seconds^20,22,23^. Maximum ICP and arterial pressure were recorded simultaneously, and total ICP was monitored by calculating the area under erectile curve (AUC) from the beginning of cavernous nerve stimulation to return of the ICP to the baseline, while MAP and the ratio of ICP/MAP were also calculated for data analysis.

### Tissue harvesting

At the completion of erectile function assessment, the rats were then sacrificed by cervical dislocation. Then the penile corpus cavernosum (below the cartilage of the glans to penile crus) was harvested and washed with phosphate buffered saline (PBS). The cavernous tissue was cut into distal (near to penile glans), medial, and proximal (near penile crus) sections and stored with liquid nitrogen. The rats were then killed by cervical dislocation.

### Western blot (WB)

Distal corpus cavernosum samples (n = 10 per group) were snipped and homogenized in RIPA lysis buffer supplemented with 1* protease inhibitor. The supernatants were collected, and protein concentrations were calculated by Coomassie brilliant blue. Protein (30 µg/lane) was loaded and separated by 10% SDS-PAGE and transferred to a polyvinylidene difluoride membrane according to standard procedures^20,21^. After blocking with 5% non-fat dry milk in TBS-T, the membrane was incubated with primary antibodies against COX-1 (1:1000, Abcam, Cambridge, MA, USA), COX-2 (1:1000, Abcam), endothelial nitric oxide synthase (eNOS) (1:1000, Abcam), and neuronal NOS (nNOS) (1:1000, Abcam) for 24 hours at 4 °C. The membranes were washed and then incubated with secondary antibody (GAPDH, 1:200, Zen BioScience Co., Ltd. Chengdu, Sichuan Province, China). Protein band densitometry was collected by a Bio-Rad ChemiDoc MP (Bio-Rad, Berkeley, CA, USA), while the band intensities were quantified by Image J software (National Institute of Health, Bethesda, MD, USA).

### Prostaglandins

6-keto PGF_1a_, thromboxane B_2_ (TXB_2_) (stable metabolites of PGI_2_ and TXA_2_, respectively), and PGE_2_ were measured by enzyme linked immunosorbent assay (ELISA) (Cayman Chemical, Ann Arbor, MI, USA) kits. The medial section of the corpus cavernosum (n = 10 for each group) was snipped and homogenized in homogenization buffer (1 mM ethylenediaminetetraacetic acid, 0.1 M phosphate buffer [pH 7.4], and 20 μg/ml indomethacin) at 4 °C to inhibit the metabolism of arachidonic acid (AA) to prostaglandins^24^. The homogenate was centrifuged at 12,000 g for 20 min at 4 °C, and the supernatant was used for measurements, according to the manufacturer’s instructions. The final prostaglandin concentrations were normalized to total protein and expressed as pg/mg protein.

### Cyclic adenosine monophosphate (cAMP), cyclic guanosine monophosphate (cGMP), and total nitric oxide (NO)

Proximal corpus cavernosum (n = 10 per group) was cut into pieces, homogenized in PBS and centrifuged (12,000 g for 20 min, at 4 °C) for supernatant collection. cAMP and cGMP were measured by ELISA kits (Nanjing Jiancheng Bioengineering Institute, Nanjing, Jiangsu Province, China) according to the manufacturer’s instructions. For total NO level, a commercially available Nitrate/Nitrite Fluorometric Assay Kit (Cayman Chemical) was used to determine the total nitrate + nitrite concentration (metabolites of NO).

### Statistical analysis

The erectile function index (ICP, AUC, ICP/MAP ratio, and MAP) and all biomarker results were analysed using GraphPad Prism 5 software (GraphPad Software, Inc., La Jolla, CA, USA). The data are shown as the mean ± SEM (standard error mean). Differences between Adult-Control and Ageing-Control groups were performed to explore the changes between adult rats and ageing rat models, while differences between Adult-Control and Adult-Aspirin groups, as well as between Ageing-Control and Ageing-Aspirin groups were compared to investigate the effects of aspirin administration on adult rats and ageing rat model, respectively. We chose the statistical method of Student’s t-test after proving Gaussian distribution using Kolmogorov-Smirnov-test, and a p-value < 0.05 was considered significant.

## Results

### Erectile function

**Figure 1:**
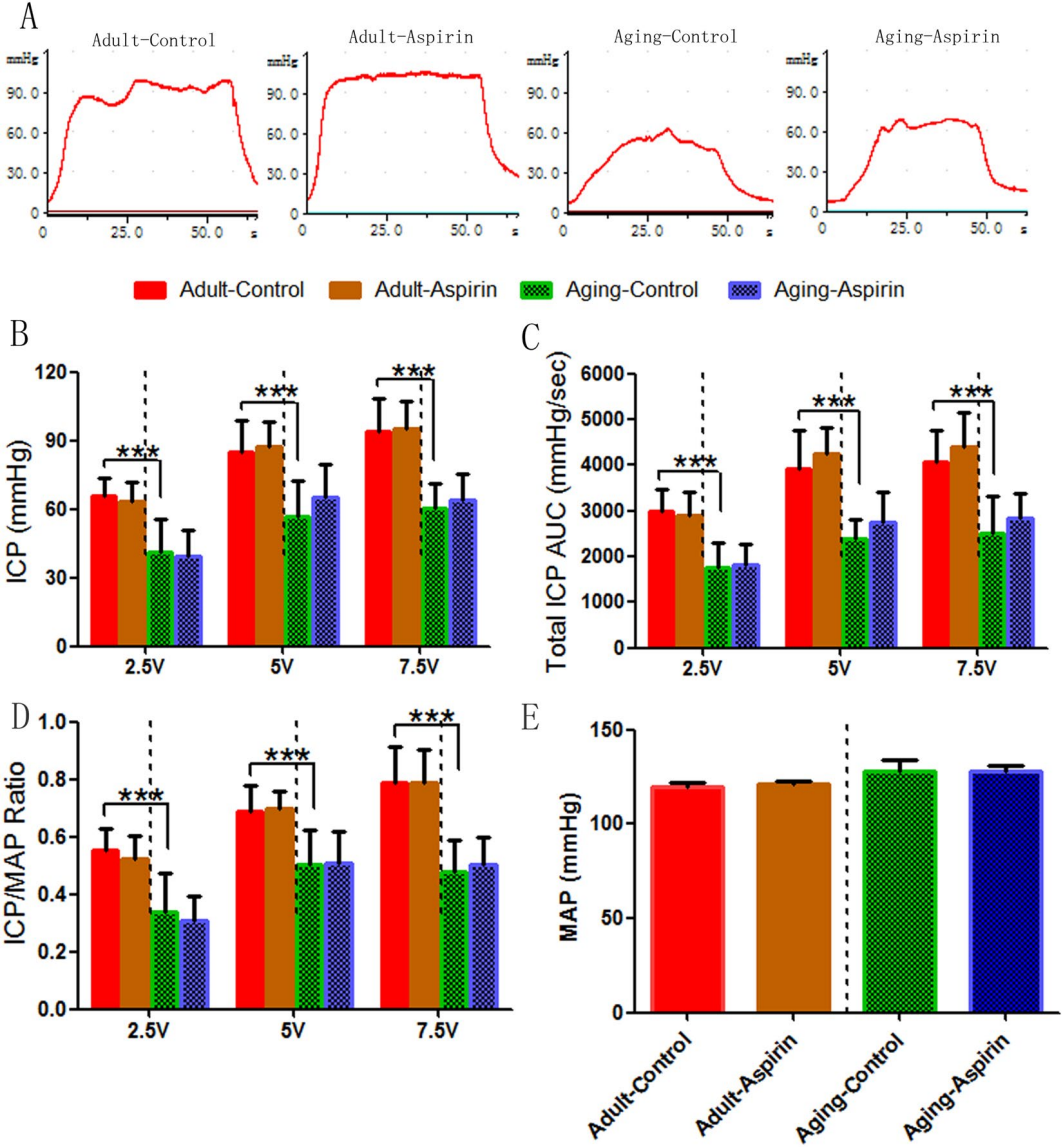
(**A**) Representative intracavernous pressure (ICP) tracings in response to cavernous nerve stimulation (5 V for 60 seconds). (**B**–**D**) Statistical analysis for voltage (2.5 V, 5 V, and 7.5 V) dependent ICP, AUC, and ICP/MAP Ratio to cavernous nerve stimulation (CNS), respectively. (**E**) Statistical analysis for MAP. The differences were analyzed with Student’s t-test between Adult-Control and Aging-Control, between Adult-Control and Adult-Aspirin, as well as between Aging-Control and Aging-Aspirin (n = 10 per group). ***< 0.0001. ICP, intracavernous pressure; AUC: total area under the ICP curves; ICP/MAP, intracavernous pressure/mean arterial pressure; MAP, mean arterial pressure.

Typical ICP tracings (5 V for 60 s) are presented in Fig. 1A. At the voltage of 5 V, our results showed that the Ageing-Control group revealed a significantly decreased ICP (p < 0.0001), AUC (p < 0.0001), and ICP/MAP ratio (p < 0.001) compared with the Adult-Control rats. However, no significant difference was observed in the ICP (p = 0.7026), AUC (p = 0.3161), and ICP/MAP ratio (p = 0.2370) between the Adult-Control and Adult-Aspirin groups. Meanwhile, aspirin also did not significantly change the ICP (p = 0.7499), AUC (p = 0.1679), or ICP/MAP ratio (p = 0.7499) between the Ageing-Control and Ageing-Aspirin groups (Fig. 1B–D).

Compared with 5 V, both lower (2.5 V) and higher (7.5 V) voltages revealed the same tendency, that was, Ageing-Control rats showed a significantly lower ICP (p = 0.0002 at 2.5 V, p < 0.0001 at 7.5 V), AUC (p < 0.0001 at 2.5 V, p = 0.0002 at 7.5 V), and ICP/MAP ratio (p = 0.0004 at 2.5 V, p < 0.0001 at 7.5 V) than Adult-Control rats. While aspirin did not significantly alter the ICP (p = 0.5782 at 2.5 V, p = 0.8241 at 7.5 V), AUC (p = 0.6381 at 2.5 V, p = 0.3528 at 7.5 V), and ICP/MAP ratio (p = 0.4464 at 2.5 V, p = 0.9738 at 7.5 V) between the Adult-Control and Adult-Aspirin groups, it also did not significantly change the ICP (p = 0.7197 at 2.5 V, p = 0.4371 at 7.5 V), AUC (p = 0.7736 at 2.5 V, p = 0.3000 at 7.5 V), and ICP/MAP ratio (p = 0.5885 at 2.5 V, p = 0.5691 at 7.5 V) between the Ageing-Control and Ageing-Aspirin groups (Fig. 1B–D).

In addition, Ageing-Control rats presented a slightly higher MAP (p = 0.2139) than Adult-Control rats, while no significant difference was found between Adult-Control and Adult-Aspirin (p = 0.5484), as well as between Ageing-Control and Ageing-Aspirin (p = 0.9669) groups (Fig. 1E).

### COX-1/2 protein expression

**Figure 2:**
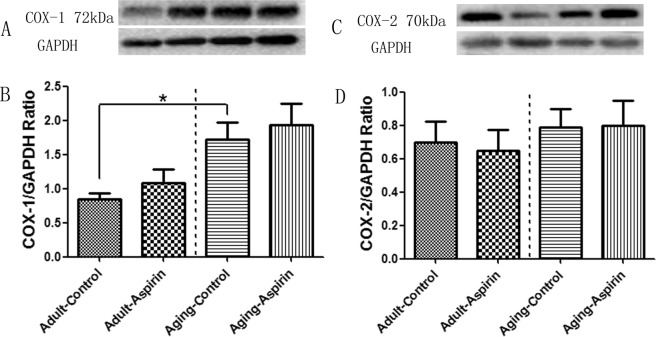
(**A**,**C**) Representative cyclooxygenase-1 (COX-1) and COX-2 bands in WB analysis (selected from gel 1 and 5 in the Supplementary File, respectively). (**B**,**D**) Statistical analysis for COX-1 and COX-2, respectively; the differences were analyzed with Student’s t-test between Adult-Control and Aging-Control, between Adult-Control and Adult-Aspirin, as well as between Aging-Control and Aging-Aspirin (n = 10 per group). *<0.05.

In WB analysis, Ageing-Control rats exhibited significantly increased COX-1 expression compared to Adult-Control rats (p = 0.0027); however, this difference was not observed between the Adult-Control and Adult-Aspirin groups (p = 0.2944), or between the Ageing-Control and Ageing-Aspirin rats (p = 0.5967) (Fig. 2A,B). In addition, no significant difference was found in terms of COX-2 levels (p = 0.8297) (Fig. 2C,D).

### Prostaglandins-cAMP pathway

**Figure 3:**
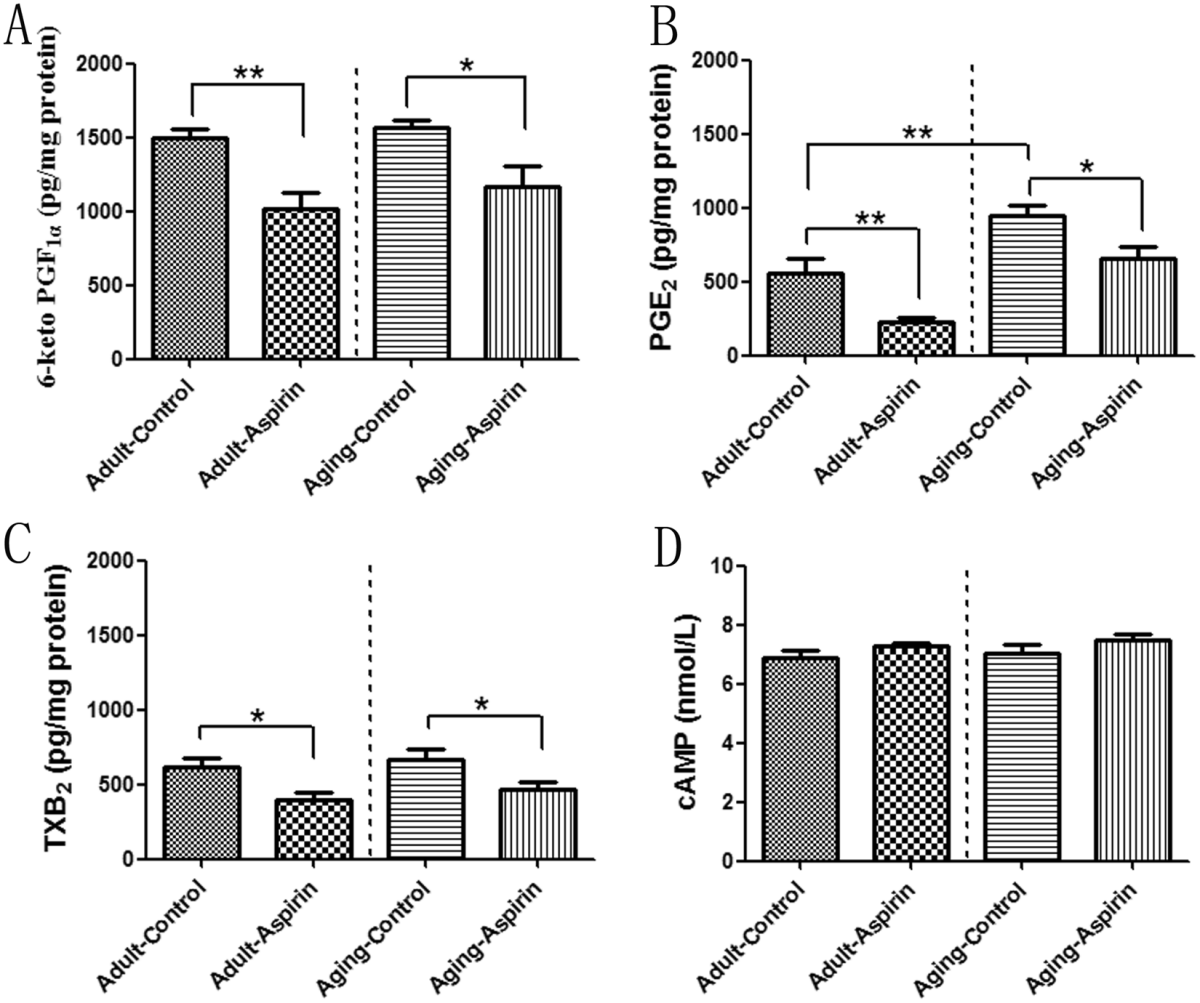
(**A**–**D**) Statistical analysis for 6-keto PGF_1a_, PGE_2_, thromboxane B_2_ and cAMP levels; the differences were analyzed with Student’s t-test between Adult-Control and Aging-Control, between Adult-Control and Adult-Aspirin, as well as between Aging-Control and Aging-Aspirin (n = 10 per group). *<0.05, **<0.01.

As the results of ELISA analysis show, Ageing-Control rats revealed significantly increased PGE_2_ levels (p = 0.0057) compared to Adult-Control rats; however, no significant difference was found for 6-keto PGF_1a_ (p = 0.3713) and TXB_2_ (p = 0.6481) between the two groups. For the adult groups, aspirin significantly decreased the 6-keto PGF_1a_ (p = 0.0019), PGE_2_ (p = 0.0080), and TXB_2_ (p = 0.0113) levels compared with the control group. The aspirin inhibitory effects on 6-keto PGF_1a_ (p = 0.0152), PGE_2_ (p = 0.0163), and TXB_2_ (p = 0.0405) were also observed in the ageing groups (Fig. 3A–C). Moreover, there was no significant difference in cAMP concentrations among groups (Fig. 3D).

### NOS-NO-cGMP pathway

**Figure 4:**
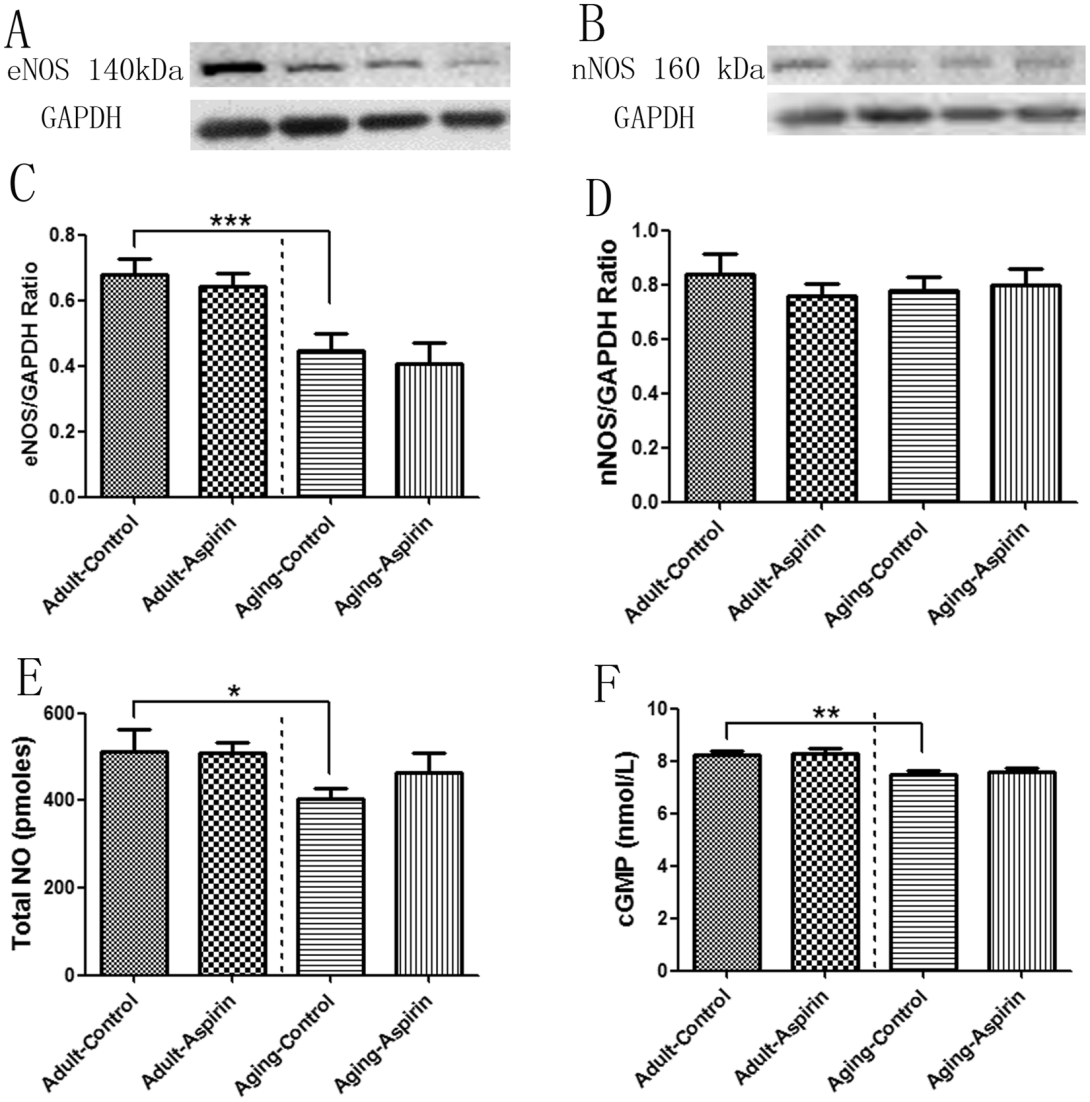
(**A**,**B**) Representative eNOS and nNOS bands in WB analysis (selected from gel 1 and 5 in Supplementary File, respectively). (**C**,**D**) Statistical analysis for eNOS and nNOS for protein expression from WB. (**E**,**F**) Statistical analysis for total NO and cGMP, respectively; the differences were analyzed with Student’s t-test between Adult-Control and Aging-Control, between Adult-Control and Adult-Aspirin, as well as between Aging-Control and Aging-Aspirin (n = 10 per group). *<0.05, **<0.01, ***<0.0001.

Ageing-Control group presented significantly decreased eNOS protein expression (p = 0.0056) compared with the Adult-Control group based on WB analysis. However, no significant difference was found between the Adult-Control and Adult-Aspirin (p = 0.6073) groups, or between the Ageing-Control and Ageing-Aspirin (p = 0.6458) groups. Moreover, there was no significant difference in nNOS expression (p = 0.7880) between the groups (Fig. 4A,B).

Ageing-Control rats had lower total NO (p = 0.0394) and cGMP (p = 0.0026) levels than Adult-Control rats. However, aspirin did not significantly reduce the concentrations of total NO (p = 0.9427) and cGMP (p = 0.8765) between the Adult-Control and Adult-Aspirin groups. For the ageing groups, aspirin also did not change the total NO (p = 0.2291) and cGMP (p = 0.7297) levels (Fig. 4C,D).

## Discussion

ED is a natural consequence of ageing, while a survey from the US National Health and Nutrition Examination revealed that the incidence increased from 8.2% in men aged 40–49 years to 77.5% in those aged over 75 years^11,25^. This can be mainly attributed to endothelial dysfunction^2,3,5,7,12^ and reduced NOS protein^26^. In Fig. 1B–D, Ageing-Control rats exhibited significantly decreased ICP, AUC, and ICP/MAP ratios (at all voltages of 2.5 V, 5 V, and 7.5 V) compared to Adult-Control rats, which confirmed decreased erectile function with age. Meanwhile, although with constant nNOS protein expression, Ageing-Control rats showed lower eNOS protein expression, and total NO and cGMP levels than Adult-Control rats (Fig. 4C,E,F), which might explain the age-dependent decreased erectile function. In addition, COX-1 protein expression was significantly higher in Ageing-Control rats than in Adult-Control rats; however, this difference was not found in COX-2 protein expression.

It has been reported that basal release of prostaglandins exhibited protective roles in many pathophysiological conditions, including normal penile erection^6^. PGI_2_ binds to the endothelial PGI receptor (IP) to activate the Gs protein-coupled receptor, which stimulates adenylyl cyclase to produce cAMP. The elevated cAMP then causes smooth muscle relaxation and induces penile erection^17^. PGE_2_ exerts a similar relaxation effect by combining PGE receptors (EP2/4)^8,16^. As vasoactive prostaglandins were formed via active COX-1/2 pathways, the release of vasodilator prostaglandins was impaired by insulin resistance, leading to enhanced vasoconstriction and blunted endothelium-dependent vasodilation^27^. Thus, the study revealed that indomethacin and diclofenac, the other COX inhibitors that reduced relaxant agents of PGI_2_ and PGE_2_^4,5,8^, adversely affected erectile responses in rats^8^. As a consequence, the authors believed that the most popular aspirin should impair normal penile erection as well.

However, our study showed that although aspirin reduced prostaglandin production in both adult rats and the ageing rat model (Fig. 3A–C), it did not change erectile function, as demonstrated by the ICP, AUC, and ICP/MAP ratios (at all voltages of 2.5 V, 5 V, and 7.5 V) (Fig. 1B–D), while aspirin had no impact on the downstream cascade of cAMP (Fig. 3D). This was in accordance with previous basic^28^ and clinical^2,3,18^ studies. They insisted and explained that penile hypercoagulability induced by TXA_2_ plays a key initiating role in penile vascular changes to reduce penile erection^8,29^; however, contractive and harmful of TXA_2_ was also inhibited by aspirin when relaxant and protective agents of PGI_2_ and PGE_2_ were reduced^16,17,29^. This might explain why aspirin did not impair erectile function.

In addition, there was a broad range of cellular responses in thromboxane receptor (TP) signalling. TPα stimulated adenylyl cyclase to produce cAMP, whereas TPβ reduced it^30,31^. Such counteracting effects occur in other receptors, such as PGE receptor (EP)2/4, which increased cAMP, but EP3 decreased it^32^. This might contribute to the unchanged cAMP level^33^ in corpus cavernosum even when aspirin blocked prostaglandin production, and it also provided further evidence that aspirin does not influence erectile function.

In fact, NO synthesized by NOS isoforms is the principal neurotransmitter in maintaining erections^7,34,35^. However, the interaction between prostaglandins and NO signalling pathways occurs on multiple levels, which requires further investigation^6,17^.

Studies revealed that PGIs increased eNOS levels^17^ while repeated PGE_1_ injection enhanced constitutive NOS isoforms (eNOS and nNOS) by stimulating prostaglandin production^8,36^, which could promote NO accumulation and strengthen erectile response to nerve stimulation^8,36^. By inhibiting prostaglandin production, aspirin or indomethacin markedly decreased NOS activity and suppressed NO concentration^6–8^ to decrease penile erectile function^7,8,16^. Therefore, aspirin could reduce erectile function by impairing the eNOS/nNOS pathway.

In contrast, some researchers believed that aspirin had the ability to restore the impaired NOS isoforms^7,37^ to increase NO bioavailability^7^. Thus, they claimed that aspirin or indomethacin could promote endothelial-dependent relaxation of the corpus cavernosum^7,35^ and improve impaired erectile function^7,34,38^.

However, both our adult rats and ageing rat model demonstrated that aspirin neither inhibited eNOS/nNOS protein expression nor reduced total NO levels. It also did not change the downstream cGMP concentrations. The constant NOS-NO-cGMP pathways were in accordance with ICP, AUC, and ICP/MAP indices, which verified that long-term aspirin administration had beneficial effects on erectile function. This was also consistent with previous clinical studies^2,3,18,39,40^.

In addition, although they share the similar pharmacological action of COX inhibition, aspirin, indomethacin, and diclofenac should be considered for a separate study on their effects on erectile processes, considering their diverse actions affecting smooth muscle tone, NO production, and blood coagulation^8^.

Our study has several limitations. First, we showed decreased prostaglandins and unchanged NO levels; however, this relationship has been controversial, and we were not able to clarify it^6,17^. Second, we chose an ageing rat model to investigate whether aspirin would change erectile function; however, the ageing model may not accurately represent all processes of ED progression, such as diabetes mellitus, dyslipidaemia, or other disease conditions. Finally, although all rats underwent ICP measurements, it was worth noting that ICP measurement activates eNOS/nNOS protein expression and cAMP/cGMP concentrations; thus, the subsequent biomarker detection might have been influenced.

## Conclusion

From the results of adult rats and the ageing rat model, although aspirin administration decreased prostaglandin levels, it did not strengthen or weaken erectile function. This is also in accordance with recent clinical studies that claim that aspirin usage has no influence on erectile function.

## Supplementary information

Supplementary File
