# Supplementary material for: Long-Term Aspirin Administration Has No Effect on Erectile Function: Evidence from Adult Rats and Ageing Rat Model
Source: Sci Rep. 2019 May 28;9:7941. doi: 10.1038/s41598-019-44386-x (PMC6538637; doi:10.1038/s41598-019-44386-x)

Long-Term Aspirin Administration Has No Effect on Erectile Function: Evidence from Adult Rats and Ageing Rat Model

Tao Li [1, 2, †], Changjing Wu [1, †], Fudong Fu [1], Wenfeng Xiong [1], Feng Qin [1], Jiuhong Yuan [1, 2, *]

[1]: The Andrology Laboratory, West China Hospital, Sichuan University, Chengdu, Sichuan, China

[2]: Department of Urology, West China Hospital, Sichuan University, Chengdu, Sichuan, China

[†] These authors contributed equally to this study and should share the co-first author.

ad-C: Adult-Control
ad-A: Adult-Aspirin
ag-C: Aging-Control
ag-A: Aging-Aspirin
No. 6 rats in each group.

1. eNOS and COX-1 bands in WB analysis was selected from Gel 1 (marked with red fonts) (n=10 per group).

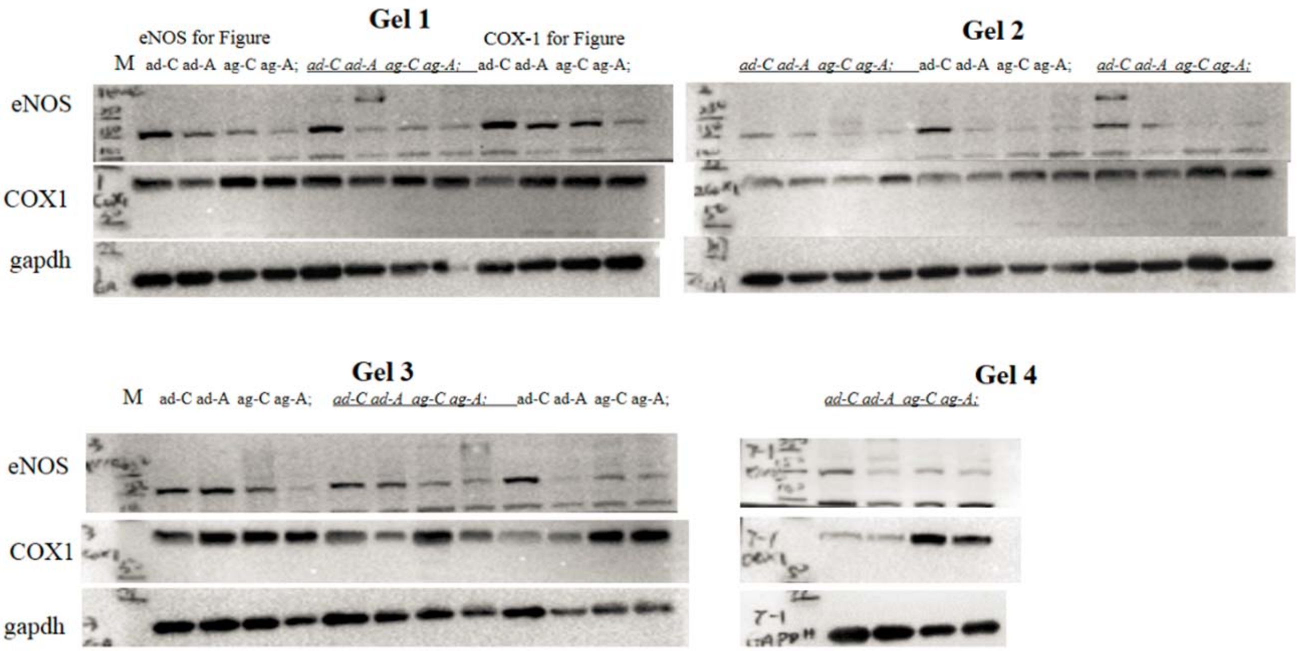

2. nNOS and COX-2 bands in WB analysis were selected from gel 5, respectively (marked with red fonts) (n=10 per group).

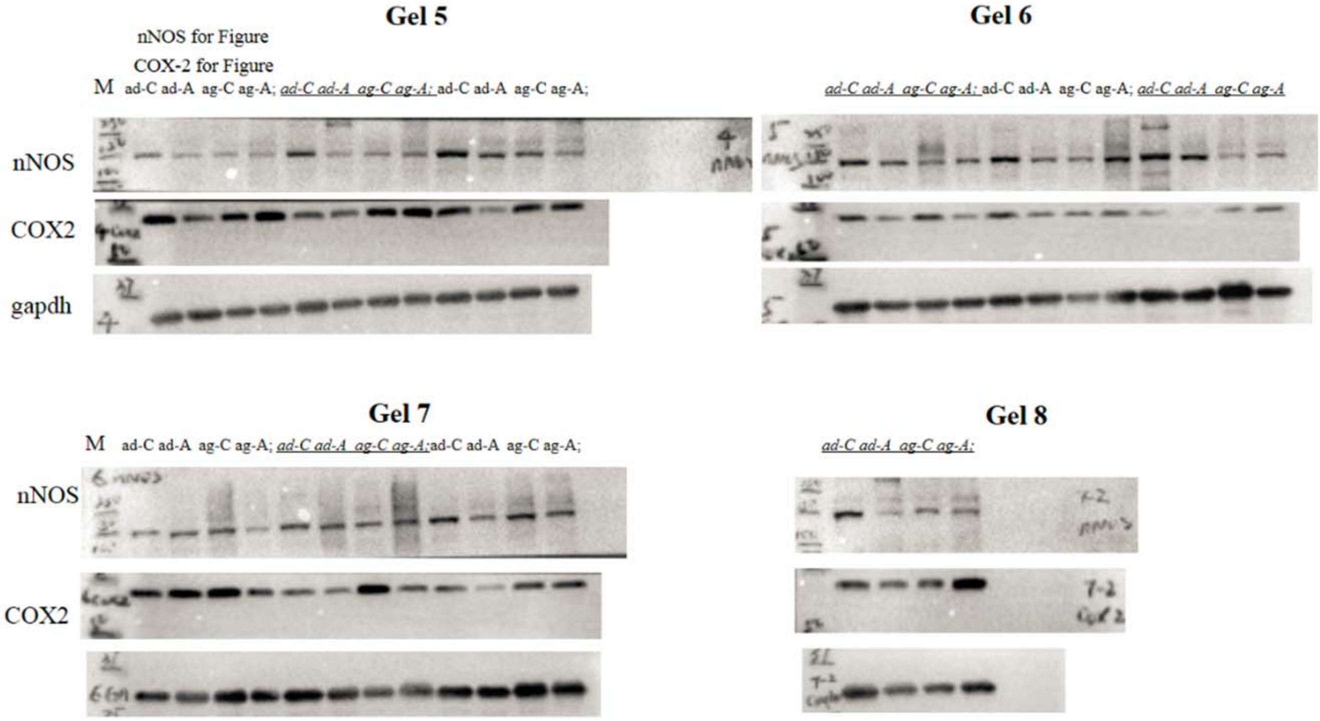

Supplement: Supplementary file 1 — Supplementary File [file 41598_2019_44386_MOESM1_ESM.pdf]
